# Does a “Cushion Effect” Really Exist? A Morphomic Analysis of Vulnerable Road Users with Serious Blunt Abdominal Injury

**DOI:** 10.3390/healthcare9081006

**Published:** 2021-08-06

**Authors:** Yu-San Tee, Chi-Tung Cheng, Chi-Hsun Hsieh, Shih-Ching Kang, Chih-Yuan Fu, Brian A. Derstine, Grace L. Su, Stewart C. Wang

**Affiliations:** 1 Division of Trauma and Emergency Surgery, Department of Surgery, Linkou Chang Gung Memorial Hospital, No. 5, Fuxing St., Guishan Dist., Taoyuan City 33302, Taiwan; b9402011@cgmh.org.tw (Y.-S.T.); atong89130@gmail.com (C.-T.C.); hsieh0818@cgmh.org.tw (C.-H.H.); sckang@cgmh.org.tw (S.-C.K.); 2 College of Medicine, Chang Gung University, Taoyuan City 33302, Taiwan; 3 Morphomic Analysis Group, University of Michigan, Ann Arbor, MI 48109, USA; bderstin@med.umich.edu (B.A.D.); gsu@med.umich.edu (G.L.S.); stewartw@med.umich.edu (S.C.W.); 4 Department of Internal Medicine, University of Michigan, Ann Arbor, MI 48109, USA; 5 Department of Medicine, VA Ann Arbor Healthcare System, Ann Arbor, MI 48105, USA; 6 Division of Acute Care Surgery, University of Michigan Medical School, Ann Arbor, MI 48109, USA

**Keywords:** cushion effect, obesity, subcutaneous fat, vulnerable road user (VRU), abdominal injury

## Abstract

*Introduction:* The severity of injury from motor vehicle crashes (MVCs) depends on complex biomechanical factors, and the bodily features of the injured person account for some of these factors. By assuming that vulnerable road users (VRUs) have limited protection resulting from vehicles and safety equipment, the current study analyzed the characteristics of fat distribution measured by computed tomography (CT) imaging and investigated the existence of a “cushion effect” in VRUs. *Materials and Methods:* This retrospective study enrolled 592 VRUs involved in MVCs who underwent CT scans. Visceral fat area and subcutaneous fat cross-sectional area were measured and adjusted according to total body area (TBA) and are presented as the visceral fat ratio and the subQ fat ratio (subcutaneous fat ratio). Risk factors for serious abdominal injury (maximum abbreviated injury scale (MAIS_abd_ ≥ 3)) resulting from MVCs were determined by univariate and multivariate analysis. *Results:* MAIS_abd_ ≥ 3 was observed in 104 (17.6%) of the patients. The subQ fat ratio at the L4 vertebral level was significantly lower in the MAIS_abd_ ≥ 3 group than in the MAIS_abd_ < 3 group (24.9 ± 12.0 vs. 28.1 ± 11.9%; *p* = 0.015). A decreased L4 subQ fat ratio was associated with a higher risk for MAIS_abd_ ≥ 3 in multivariate analysis (odds ratio 0.063; 95% CI 0.008–0.509; *p* = 0.009). *Conclusion:* The current study supported the “cushion effect” theory, and protection was apparently provided by subcutaneous fat tissue. This concept may further improve vehicle and safety designation in the future.

## 1. Background

Motor vehicle crashes (MVCs) are a leading cause of death in the young population [1]. Injury severity from a crash depends on complex biomechanical factors, such as vehicle type, velocity of crash, type of impact, and safety equipment. Although crash fatalities improved after the development of and requirement for safety equipment [2], an individual’s bodily features are an unchangeable component in MVCs.

Body mass index (BMI) is the most discussed assessment of an individual’s body size. It is a simple but also an indirect measurement of physical status. One of the most popular bodily features, obesity, is characterized as excess fat accumulation. It is defined as a BMI ≥ 30 kg/m^2^ by adopting National Institutes of Health (NIH) and World Health Organization (WHO) guidelines [3,4]. It has been widely discussed in the trauma literature and studies have shown that obesity is associated with a higher risk for post-trauma complications and mortality [5,6,7,8,9,10,11,12,13,14,15,16,17,18,19].

Although a greater body mass carries higher momentum, the injury pattern of an obese occupant diverges from those with normal weight or those who are underweight in the crash. Arbabi et al. introduced the “cushion effect” in 2003, suggesting that increased abdominal adiposity provides a “cushion” for abdominal trauma during the injury event [20]. Wang et al. and Fu et al. also found that obesity protects patients from severe abdominal injury [21,22]. A computational study found that an obese dummy model had increased risks for head, thorax, and lower extremity injury due to greater torso and pelvic excursions, while increased subcutaneous fat may cushion subjects from abdominal injury [23]. However, the establishment of the theory was based on limited data from car occupants [6,21], and the conclusions remain controversial [24,25,26]. Distinct from car occupants, vulnerable road users (VRUs) lack protection from metallic shells and safety equipment [27]. The results of the collision may be significantly altered due to different physical characteristics. Hence, understanding the bodily features of VRUs in crashes is crucial and may provide clues for clinical judgements and post-trauma outcomes.

However, the ease of use of BMI does not overcome its inability to distinguish between bone, muscle mass, and fat tissue. Regardless of the fact that the development of modern computed tomography (CT) imaging and software enables the precise measurement of body composition, the exact mechanism and interaction between the human body and collision is still poorly understood. To further investigate the impact of bodily characteristics during an MVC, the current study was performed by assessing VRUs with limited abdominal protection during the collision. We analyzed the characteristics of body fat distribution in VRUs and hypothesized the existence of a “cushion effect” in blunt abdominal trauma.

## 2. Materials and Methods

### 2.1. Data Collection

The data from May 2008 to December 2016 were collected from the trauma registry of our institution. Individuals who were involved in MVCs were included if they were older than 16 years old, had an abdominal multidetector helical computed tomography (MDCT) scan performed primarily for trauma indications, and were admitted to the ward or intensive care unit (ICU). Indications for an MDCT scan included a positive Focus Assessment with Sonography in Trauma (FAST), an abnormal finding in physical examination of the abdomen or pelvis, an abnormal chest or pelvic X-ray, an unconscious occupant with blunt torso injury, or clinical judgment. All occupants underwent MDCT scans if they were hemodynamically stable either with or without resuscitation. Occupants with incomplete medical records or missing body height or weight measurements were excluded.

Data on the following variables were collected: age, sex, height, weight, BMI, Glasgow coma scale (GCS), and vital signs at the emergency department. Vehicle data, including vehicle type and safety equipment, were collected. The VRUs are defined as road users that do not have shell protection during an MVC, who consist of pedestrians, bicyclists, and motorcyclists [28]. Injury severity was assessed using the Abbreviated Injury Scale (AIS) and Injury Severity Score (ISS). The AIS ranges from 0 to 6. The Maximum Abbreviated Injury Scale (MAIS) of each body region was then determined.

Hemodynamic instability was defined according to at least one episode of systolic pressure less than 90 mmHg upon emergency admission. A MAIS of 3–6 (MAIS ≥ 3) was defined as serious injury in each body region.

### 2.2. Morphomic Variables

**Figure 1:**
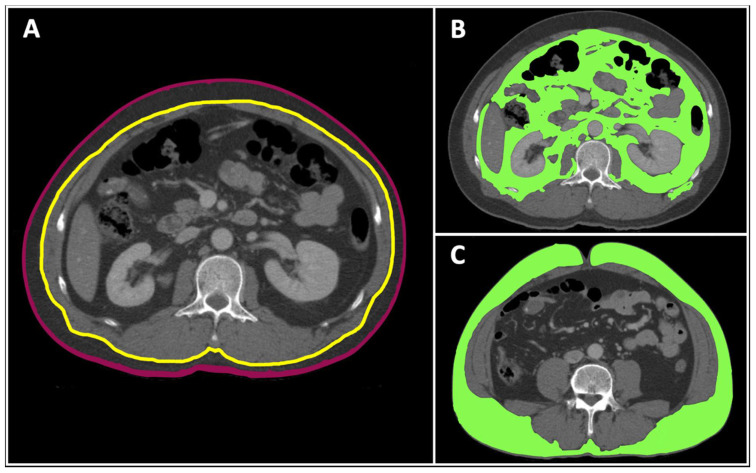
This figure demonstrates the morphomic variable measurements of a 53-year-old male motorcyclist by a CT scan. Total body area was measured by the cross-sectional area inside the purple line (**A**), whereas the visceral fat area (at the L2 vertebral level) was represented by the area inside the fascia (yellow line) meeting fat density thresholds (**B**), and the subcutaneous fat area (at the L4 vertebral level) was represented by the area between the skin and fascia meeting fat density thresholds (**C**).

CT images were assessed with analytic morphomics, which has been previously described [29,30]. The total body area (TBA), visceral fat area, and subcutaneous fat area were measured from the T9 to L5 vertebrae. The results are given in square centimeters (cm^2^) (Figure 1). Because individuals’ body sizes vary, the visceral fat area and subcutaneous fat area were adjusted according to the TBA at the corresponding vertebral level before further analyses. The results are presented as the visceral and subQ fat ratios (subcutaneous fat ratio, %).

- TBA: the cross-sectional area of the body.
- Visceral fat area: the cross-sectional area within the fascia with fat density thresholds between −205 and −51 Hounsfield units (HU).
- Subcutaneous fat area: the cross-sectional area between skin and fascia with fat density thresholds between −205 and −51 HU.
- Visceral fat ratio (%) = $\frac{\mathrm{Visceral}\;\mathrm{fat}\;\mathrm{area}\;\left({\mathrm{cm}}^{2}\right)}{\mathrm{TBA}\;\left({\mathrm{cm}}^{2}\right)}$ × 100%.
- SubQ fat ratio (%) = $\frac{\mathrm{Subcutaneous}\;\mathrm{fat}\;\mathrm{area}\;\left({\mathrm{cm}}^{2}\right)}{\mathrm{TBA}\;\left({\mathrm{cm}}^{2}\right)}$ × 100%.

### 2.3. Statistical Analysis

Descriptive statistics were calculated for the cohort. Categorical data are presented as numbers and percentages and were compared using the chi-square test. Continuous variables are presented as the mean with standard deviation (SD) or median with interquartile range (GCS, MAIS, and ISS). Continuous variables were compared using Student’s *t* test or the Mann–Whitney U test (for non-normally distributed data). A multivariable logistic regression model was performed to examine the relationship between serious abdominal injury and the subcutaneous fat ratio, adjusting for patient and crash characteristics.

A significance level of α = 0.05 was used. All analyses were performed using IBM SPSS statistics 25.0 (IBM Corporation, Armonk, NY, USA). Microsoft Excel (V16.19) was used for data entry and to create associated figures.

## 3. Results

Between May 2008 and December 2016, 592 VRUs involved in MVCs underwent abdominal CT scans primarily for trauma evaluation. The demographics of these studied patients are summarized in Table 1. Their mean age and mean BMI were 38.7 ± 18.1 years and 24.2 ± 4.7 kg/m^2^, respectively. Of these patients, 59 (10.0%) were pedestrians, and 533 (90.0%) patients were bicyclists or motorcyclists involved in an MVC. A total of 98 patients did not use safety equipment, while the other 494 wore helmets. Upon arrival at our emergency department (ED), 73 individuals (12.3%) had unstable hemodynamics. The median GCS was 15, and the median ISS was 18. Among these individuals, 156 (26.4%) had serious (MAIS ≥ 3) head injuries, 269 (45.4%) had serious thoracic injuries, 104 (17.6%) had serious abdominal injuries, and 175 (29.6%) had serious limb injuries. Overall mortality in the cohort was 5.4%, with a mean length of hospital stay of 16.3 ± 15.7 days and a mean length of ICU stay of 5.1 ± 7.9 days. The distribution of BMI and BMI classifications of the enrolled VRUs is shown in Supplementary Figure S1 and Table S1.

**Figure 2:**
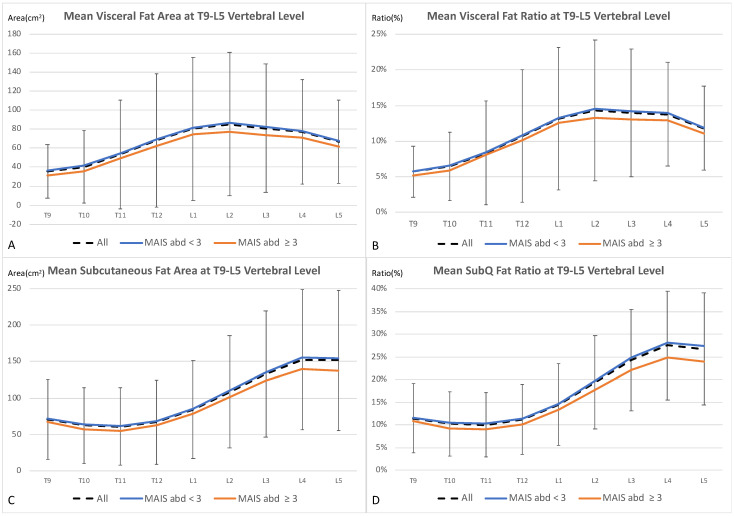
(**A**–**D**). Mean visceral fat area (**A**) and subcutaneous fat area (**C**) from the T9-L5 vertebral level are shown. The visceral fat ratio (**B**) and subQ fat ratio (**D**) were obtained. The standard deviation of all VRUs was represented in black error bars. While visceral fat area and ratio peaked at the L2 level, subcutaneous fat area and ratio peaked at the L4 level. Vulnerable road users with serious abdominal injury (MAIS_abd_ ≥ 3, represented in blue lines) had lower fat areas and ratios than those with minor injury (MAIS_abd_ < 3, brown lines).

The fat areas from the T9 to L5 vertebral levels are summarized in Supplementary Table S2 and Figure 2. Mean visceral and subcutaneous fat areas (Figure 2A,C) were adjusted according to TBA at corresponding levels to obtain fat ratios (Figure 2B,D). The mean visceral fat area and ratio gradually increased from the thoracic vertebral level and peaked at the L2 vertebral level, while the mean subcutaneous fat area and ratio were lowest at the T11 level and peaked at the L4 level. Hence, the L2 visceral fat ratio and the L4 subQ fat ratio were used for further analyses in the cohort. The mean L2 visceral fat ratio was 14.3 ± 9.9%, while the mean L4 subQ fat ratio was 27.5 ± 12.0% in the cohort. While all fat areas and fat ratios from the T9 to L5 level were lower in VRUs with MAIS_abd_ ≥ 3, the differences were only significant in subQ fat ratios at the L3, L4, and L5 vertebral levels (Supplementary Table S2).

Table 2 shows a comparison of the characteristics of VRUs with and without serious abdominal trauma (MAIS_abd_ ≥ 3). There were no significant differences in age, BMI, sex, vehicle, or safety equipment usage. VRUs with serious abdominal injury did not have a longer length of stay (LOS), and they did not have a higher mortality rate according to the analysis. However, the MAIS_abd_ ≥ 3 group had a longer ICU LOS (6.5 ± 7.8 vs. 4.8 ± 7.9 days, *p* = 0.038). Notably, VRU with a lower mean L4 subQ fat ratio was more likely to have serious abdominal trauma (24.9 ± 12.0 vs. 28.1 ± 11.9%, *p* = 0.015), although the BMI was similar.

**Figure 3:**
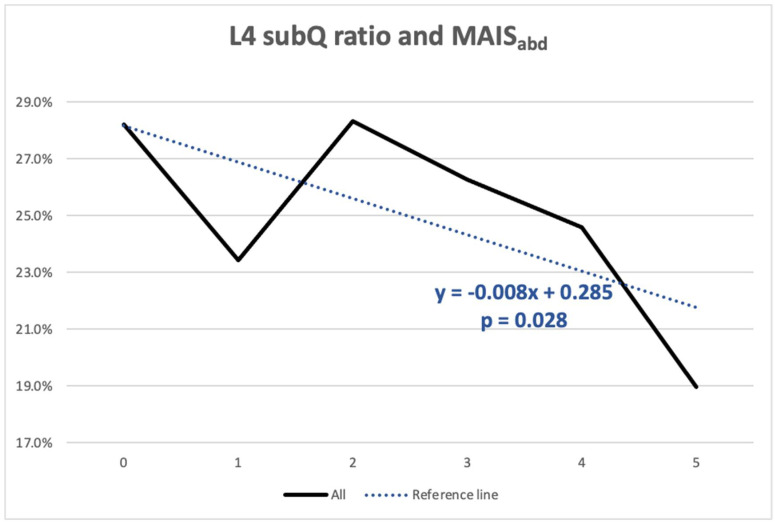
This figure shows that the mean L4 SubQ fat ratio is inversely correlated with abdominal MAIS (*p* = 0.028).

Table 3 shows the importance of the variables in the logistic regression model for serious abdominal trauma. When combining the demographic, vehicle, and morphomic variables, the results indicated that the risk of serious abdominal trauma increased as the L4 subQ ratio decreased (OR 0.063; 95% CI 0.008–0.509; *p* = 0.009). BMI and L2 visceral fat ratio were not independent predictors of serious abdominal injury. The correlations between the L4 subQ ratio and MAIS_abd_ are shown in Figure 3. Interestingly, there was a significant trend showing that abdominal injury severity increased as the L4 subQ fat ratio decreased (β = −0.008, *p* = 0.028).

## 4. Discussion

The prevalence of obesity is increasing worldwide and has been well studied in terms of the associated cardiovascular and metabolic risks [31,32,33]. While the literature suggests that the obese trauma population has poorer outcomes than the normal weight trauma population [10,14,15], some studies have indicated that increased abdominal adiposity may protect individuals from serious abdominal trauma during frontal MVCs [20].

Adipose tissue can be stored beneath the skin, around organs, and within bone marrow or muscle. In addition to its role in energy storage and endocrine function [34], it also provides protective padding to organs during collision. Although BMI has been widely used as a simple indirect measurement of body mass, it does not distinguish body components. By analyzing body composition from CT scans, Wang et al. suggested that increased subcutaneous fat protects females from serious abdominal injury during frontal crashes, but they identified a less significant trend in males [21]. However, the small cohort and fat measurement conducted solely according to fat depth at the L4 vertebral level may not provide conclusive evidence for the “cushion” phenomenon. In addition, the literature discussing the protective mechanism of fat cushion in trauma patients was limited to car occupants in frontal crashes [20,25,26]. Because car occupants are often protected by a metallic chassis, seatbelt, or airbag assembly, the current study enrolled VRUs who have the least protection from the vehicle and safety equipment. By describing the distribution of torso subcutaneous and visceral fat measured by CT images, the current study found solid evidence that increased subcutaneous fat tissue lowers the risk for serious abdominal injury in VRUs during MVCs, supporting the concept of the “cushion” theory in a wider population than was previously thought.

The utility of CT-measured visceral and subcutaneous fat tissue in the trauma population grew because of the development of CT imaging and software. A prospective study by Shashaty et al. reported that increased L4-L5 visceral fat increased prevalence of acute kidney injury in 327 trauma patients [35], though Collier et al. found that increased visceral fat at L1 level was not associated with trauma outcomes [36]. Another smaller study, which analyzed 57 patients with abdominal trauma who needed surgical intervention, reported that the visceral fat area, subcutaneous fat area, and visceral/subcutaneous fat ratio did not demonstrate a significant finding in post-operative outcomes [37]. In contrast to visceral fat tissue, abdominal subcutaneous fat tissue is less discussed in traumatology [25,26,38,39]. The current study demonstrated that the visceral fat area peaks at the L2 vertebral level and that the subcutaneous fat area peaks at the L4 vertebral level. As fat area varies with body size, it was adjusted according to the TBA for further analysis. Interestingly, subcutaneous fat was found to be a stronger protective factor against serious abdominal trauma during MVCs than visceral fat, showing that subcutaneous fat tissue is more likely to disperse forces during crashes.

As a growing issue worldwide [40], obesity has been discussed in trauma populations for decades. BMI classification is the most commonly used parameter in discussing the impact of obesity on trauma outcomes. Similar to the literature, obese VRU in the cohort had a significantly higher mortality rate (14.1%) than the non-obese group (Supplementary Table S1). However, different BMI groups did not account for significant differences in serious abdominal injury. Despite discussing trauma outcomes by using traditional BMI classifications, the current study was conducted by using CT-measured fat area and ratio as alternatives to investigate the impact of morphological features of VRU during trauma.

Viano et al. found that obese patients had a 40% higher risk of serious injury than normal weight patients. However, the abbreviated injury scales of each body region were not discussed [24]. In the current study, VRU with serious abdominal trauma had a 10–20% lower L4 subQ fat ratio than the minor abdominal injury group, and the difference was significant. As mentioned, low BMI was not an independent risk factor for serious abdominal trauma in the cohort. Despite the fact that momentum increases as mass increases, the results revealed that abdominal injury severity might differ due to different quantities and distributions of subcutaneous fat under conditions of similar momentum.

The current cohort was from a level I trauma center in Taiwan, with a median ISS of 17.5 and an overall in-hospital mortality rate of 5%, which was in line with a previous report [8,14,41,42]. Although an increased L4 subQ ratio protected VRU from serious abdominal trauma, it was not associated with mortality reduction, which could be explained by the low correlation between abdominal injury severity and mortality [43]. However, VRU with serious abdominal trauma had a significantly longer ICU LOS.

Finally, it is important to realize that the current study was limited due to its retrospective nature. A lower rate of obesity and morbid obesity in Asia also limited the ability to demonstrate a full picture of the “cushion effect”. Furthermore, vital organs such as the liver, spleen, and kidneys are located between the T11 and L2 vertebral levels. An analysis of fat area at a single vertebral level may not be conclusive.

## 5. Conclusions

The advancement of CT imaging provides a chance to inspect the complex interaction between the human body and injury. The results of the current study supported the existence of a fat cushion effect. Increased subcutaneous fat in VRU involved in MVCs is associated with lower prevalence of serious abdominal trauma. The concept may further improve the development of vehicles and safety features in the future.

## Figures and Tables

**Table 1:** Characteristics of enrolled vulnerable road users.

	*n* = 592
**Demographic Variables**	
Age (years) ^a^	38.7 ± 18.1
Sex (%)	
M	403 (68.1%)
F	189 (31.9%)
Weight (kg) ^a^	67.1 ± 15.2
Height (m^2^) ^a^	166.1 ± 9.1
BMI (kg/m^2^) ^a^	24.2 ± 4.7
**Vehicle Variables**	
Vehicle type (%)	
Pedestrian	59 (10.0%)
Bicyclist/Motorcyclist	533 (90.0%)
Safety equipment type (%)	
No safety equipment	98 (16.6%)
Helmet	494 (83.4%)
**Injury Severity**	
Unstable hemodynamics (*n*, %)	73 (12.3%)
Coma scale (GCS) ^b^	15 (13–15)
ISS ^b^	18 (9–27)
Serious head injury (*n*, %)	156 (26.4%)
Serious thoracic injury (*n*, %)	269 (45.4%)
Serious abdominal injury (*n*, %)	104 (17.6%)
Serious limb injury (*n*, %)	175 (29.6%)
**Morphomics Variables**	
L2 visceral fat area (cm^2^) ^a^	85.33 ± 75.27
L4 subcutaneous fat area (cm^2^) ^a^	152.30 ± 96.12
L2 visceral fat ratio (%) ^a^	14.3% ± 9.9%
L4 subQ fat ratio (%) ^a^	27.5% ± 12.0%
**Outcomes**	
LOS (days) ^a^	16.3 ± 15.7
ICU LOS (days) ^a^	5.1 ± 7.9
Mortality (%)	32 (5.4%)

BMI: body mass index, GCS: Glasgow coma scale, ISS: injury severity score, subQ fat ratio: subcutaneous fat ratio, LOS: length of hospital stay, ICU LOS: length of ICU stay. ^a^ Mean ± SD. ^b^ Median (25–75% interquartile range).

**Table 2:** Univariate analysis of risk factors for serious abdominal injury (MAIS_abd_ ≥ 3) in vulnerable road users.

	MAIS_abd_ < 3	MAIS_abd_ ≥ 3	*p*-Value
	*n* = 488 (82.4%)	*n* = 104 (17.6%)	
**Demographic Variables**			
Age (years) ^a^	39.4 ± 18.2	35.7 ± 17.2	0.053
BMI (kg/m^2^) ^a^	24.3 ± 4.6	24.2 ± 5.4	0.889
Sex (%) ^#^			0.781
M	331 (67.8%)	72 (69.2%)	
F	157 (32.2%)	32 (30.8%)	
Participant type ^#^			0.076
Pedestrian	53 (10.9%)	6 (5.8%)	
Bicyclist/Motorcyclist	435 (89.1%)	98 (94.2%)	
Safety Equipment (*n*, %) ^#^			0.221
No safety equipment	85 (17.4%)	13 (12.5%)	
Helmet	403 (82.6%)	91 (87.5%)	
**Injury Severity**			
Coma scale (GCS) ^b,†^	15 (10–15)	15 (15–15)	0.002 *
Unstable hemodynamics (*n*, %) ^#^	56 (11.5%)	17 (16.3%)	0.170
ISS ^b,†^	17 (9–25)	25 (18–32)	<0.001 *
MAIS_head_^b,†^	0 (0–3)	0 (0–0)	<0.001 *
MAIS_chest_^b,†^	1 (0–3)	3 (0–4)	0.026 *
MAIS_limb_^b,†^	2 (0–3)	1 (0–2)	0.001 *
Serious head injury (*n*, %) ^#^	149 (30.5%)	7 (6.7%)	<0.001 *
Serious chest injury (*n*, %) ^#^	208 (42.6%)	61 (58.7%)	0.003 *
Serious limb injury (*n*, %) ^#^	150 (30.7%)	25 (24%)	0.174
**Morphomic Variables**			
L2 visceral fat ratio (%) ^a,!^	14.6 ± 10.0%	13.3 ± 9.0%	0.205
L4 subQ fat ratio (%) ^a,!^	28.1 ± 11.9%	24.9 ± 12.0%	0.015 *
**Outcomes**			
LOS (days) ^a,!^	15.9 ± 15.6	18.0 ± 16.3	0.223
ICU LOS (days) ^a,!^	4.8 ± 7.9	6.5 ± 7.8	0.038 *
Mortality (*n*, %) ^#^	29 (5.9%)	3 (2.9%)	0.211

BMI: body mass index, GCS: Glasgow coma scale, ISS: injury severity score, MAIS: maximal abbreviated injury scale, subQ fat ratio: subcutaneous fat ratio, LOS: length of hospital stay, ICU LOS: length of ICU stay; ^a^ mean ± SD; ^b^ median (25–75% interquartile range); ^#^ chi-squared test; ^!^ Student’s *t* test; ^†^ Mann–Whitney U test; * statistically significant.

**Table 3:** Multivariate logistic regression of risk factors for serious abdominal injury (MAIS_abd_ ≥ 3).

Variable	Coef (β)	Std Err	Odds Ratio	95% CI	*p*-Value
Intercept	−2.135	1.165	0.118		0.067
Age	−0.012	0.009	0.988	0.972–1.005	0.175
BMI	0.035	0.028	1.035	0.979–1.094	0.223
Vehicle type (pedestrian)	0.441	0.470	1.555	0.619–3.906	0.348
L2 visceral fat ratio	0.551	1.677	1.736	0.065–46.464	0.742
L4 subQ fat ratio	−2.758	1.063	0.063	0.008–0.509	0.009 *

BMI: body mass index, MAIS: maximal abbreviated injury scale, subQ fat ratio: subcutaneous fat ratio. * Statistically significant.

## Data Availability

The datasets analyzed in the current study are not publicly available due to institutional policy but are available from the corresponding author on reasonable request.

## Supplementary Materials

The following are available online at https://www.mdpi.com/article/10.3390/healthcare9081006/s1, Figure S1. The BMI distribution of vulnerable road users (VRUs) in the cohort was demonstrated, Table S1. Vulnerable road users (VRUs) were stratified by using BMI classification. There was no significant difference in the serious abdominal injury (MAIS_abd_ ≥ 3) rate between the BMI groups, but obese VRU had a significantly higher mortality rate (14.1%) than the other BMI groups (*p* = 0.012), Table S2. Mean visceral fat and subcutaneous fat areas from the T9-L5 vertebral levels are shown. Vulnerable road users with serious abdominal injury (MAIS_abd_ ≥ 3) had lower fat areas at most of the levels. After adjusting the morphomic variables according to the corresponding total body area (TBA), the fat ratio at each vertebral level is shown.

## Abbreviations

MVCs	Motor vehicle crashes
VRUs	Vulnerable road users
BMI	Body mass index
CT	Computed tomography
MDCT	Multidetector helical computed tomography
ICU	Intensive care unit
FAST	Focus Assessment with Sonography in Trauma
GCS	Glasgow Coma Scale
AIS	Abbreviated Injury Scale
MAIS	Maximum Abbreviated Injury Scale
ISS	Injury Severity Score
TBA	Total body area
SubQ fat ratio	Subcutaneous fat area
SD	Standard deviation
ED	Emergency department
