# Supplementary material for: Does a “Cushion Effect” Really Exist? A Morphomic Analysis of Vulnerable Road Users with Serious Blunt Abdominal Injury
Source: Healthcare (Basel). 2021 Aug 6;9(8):1006. doi: 10.3390/healthcare9081006 (PMC8393396; doi:10.3390/healthcare9081006)
Supplement: Supplementary file 1 [file healthcare-09-01006-s001.zip › healthcare-1311748-supplementary.pdf]

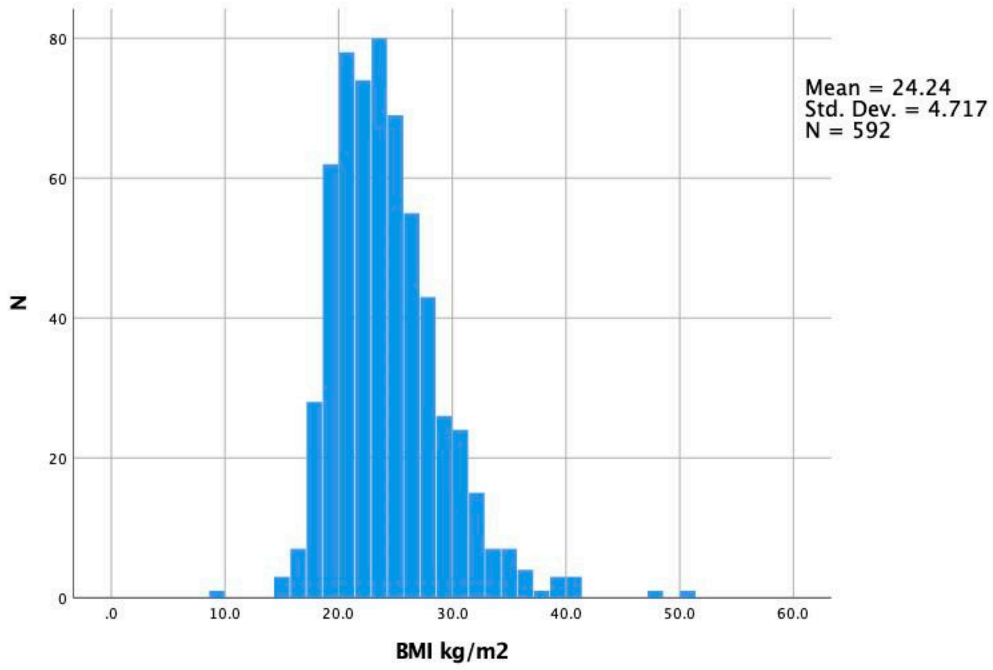

Supplementary Figure S1. The BMI distribution of vulnerable road users (VRUs) in the cohort was demonstrated.

Supplementary Table S1. Vulnerable road users (VRUs) were stratified by using BMI classification. There was no significant difference in the serious abdominal injury ($MAIS_{abd} \geq 3$) rate between the BMI groups, but obese VRU had a significantly higher mortality rate (14.1%) than the other BMI groups (p = 0.012).

BMI classification	kg/m^2	n	$MAIS_{abd} \geq 3$	Mortality rate
**Total**		592	17.6%	5.4%
**Underweight**	<18.5	39	23.1%	2.6%
**Normal**	≥18.5 to 24.9	336	17.3%	4.2%
**Overweight**	≥25.0 to 29.9	153	15.0%	5.2%
**Obese**	≥30	64	21.9%	14.1%
p value			0.505	0.012*

* Statistically significant.

Supplementary Table S2. Mean visceral fat and subcutaneous fat areas from the T9-L5 vertebral levels are shown. Vulnerable road users with serious abdominal injury ($MAIS_{abd} \geq 3$) had lower fat areas at most of the levels. After adjusting the morphomic variables according to the corresponding total body area (TBA), the fat ratio at each vertebral level is shown.

Morphomic Variables	Vertebral Level	All n = 592	$MAIS_{abd} < 3$ n = 488	$MAIS_{abd} \geq 3$ n = 104	p-value
Visceral fat area (cm²)	T9	35.65 ± 28.38	36.52 ± 29.22	31.42 ± 23.50	0.063
	T10	40.36 ± 38.35	41.30 ± 39.43	35.91 ± 32.61	0.193
	T11	53.59 ± 57.49	54.38 ± 59.15	49.92 ± 49.09	0.473
	T12	68.49 ± 70.37	69.72 ± 72.30	62.75 ± 60.52	0.360
	L1	80.63 ± 75.39	81.84 ± 77.24	74.95 ± 66.07	0.398
	L2	85.33 ± 75.27	87.14 ± 77.54	76.87 ± 63.19	0.151
	L3	80.95 ± 67.55	82.47 ± 69.08	73.83 ± 59.60	0.237
	L4	77.3 ± 54.95	78.55 ± 56.11	71.39 ± 48.98	0.228
	L5	66.56 ± 43.88	67.59 ± 45.04	61.78 ± 37.83	0.223
Subcutaneous fat area (cm²)	T9	70.66 ± 54.97	71.43 ± 54.83	66.92 ± 55.79	0.460
	T10	62.2 ± 51.69	63.40 ± 51.56	56.56 ± 52.14	0.221
	T11	60.84 ± 52.97	62.05 ± 52.85	55.14 ± 53.46	0.228
	T12	66.98 ± 57.8	68.01 ± 57.27	62.18 ± 60.29	0.351
	L1	83.75 ± 67.29	84.77 ± 66.8	78.93 ± 69.69	0.422
	L2	108.38 ± 76.76	109.89 ± 76.18	101.29 ± 79.42	0.300
	L3	133.15 ± 86.37	135.22 ± 85.81	123.44 ± 88.69	0.207
	L4	152.3 ± 96.12	154.97 ± 96.19	139.80 ± 95.28	0.144
	L5	151.77 ± 96.4	154.75 ± 96.25	137.94 ± 96.40	0.108
Visceral fat ratio (%)	T9	5.7 ± 3.6%	5.8 ± 3.6%	5.2 ± 3.3%	0.118
	T10	6.4 ± 4.8%	6.5 ± 4.8%	5.9 ± 4.5%	0.201
	T11	8.3 ± 7.3%	8.4 ± 7.4%	8.1 ± 7.0%	0.698
	T12	10.8 ± 9.3%	10.9 ± 9.4%	10.2 ± 8.7%	0.492
	L1	13.1 ± 10.0%	13.2 ± 10.1%	12.6 ± 9.5%	0.553
	L2	14.3 ± 9.9%	14.6 ± 10.0%	13.3 ± 9.0%	0.205
	L3	14.0 ± 9.0%	14.2 ± 9.1%	13.0 ± 8.5%	0.241
	L4	13.8 ± 7.3%	14.0 ± 7.3%	12.9 ± 7.1%	0.191
	L5	11.8 ± 5.9%	11.9 ± 6.0%	11.1 ± 5.4%	0.214
SubQ fat ratio (%)	T9	11.5 ± 7.6%	11.6 ± 7.6%	10.8 ± 7.4%	0.331
	T10	10.3 ± 7.1%	10.5 ± 7.2%	9.2 ± 7.0%	0.099
	T11	10.0 ± 7.1%	10.3 ± 7.1%	9.0 ± 7.0%	0.089
	T12	11.2 ± 7.7%	11.4 ± 7.6%	10.2 ± 8.0%	0.152

	L1	14.4 ± 9.0%	14.7 ± 8.9%	13.4 ± 9.4%	0.181
	L2	19.4 ± 10.2%	19.7 ± 10.1%	17.8 ± 10.6%	0.070
	L3	24.3 ± 11.1%	24.8 ± 10.9%	22.1 ± 11.5%	0.022*
	L4	27.5 ± 12%	28.1 ± 11.9%	24.9 ± 12.0%	0.015*
	L5	26.7 ± 12.3%	27.3 ± 12.2%	24.0 ± 12.4%	0.013*

* Statistically significant.
